# An Improved Grey Wolf Optimization Algorithm with Variable Weights

**DOI:** 10.1155/2019/2981282

**Published:** 2019-06-02

**Authors:** Zheng-Ming Gao, Juan Zhao

**Affiliations:** ^1^School of Computer Engineering, Jingchu University of Technology, Jingmen, Hubei 448000, China; ^2^School of Electronics and Information Engineering, Jingchu University of Technology, Jingmen, Hubei 448000, China

## Abstract

With a hypothesis that the social hierarchy of the grey wolves would be also followed in their searching positions, an improved grey wolf optimization (GWO) algorithm with variable weights (VW-GWO) is proposed. And to reduce the probability of being trapped in local optima, a new governing equation of the controlling parameter is also proposed. Simulation experiments are carried out, and comparisons are made. Results show that the proposed VW-GWO algorithm works better than the standard GWO, the ant lion optimization (ALO), the particle swarm optimization (PSO) algorithm, and the bat algorithm (BA). The novel VW-GWO algorithm is also verified in high-dimensional problems.

## 1. Introduction

A lot of problems with huge numbers of variables, massive complexity, or having no analytical solutions were met during the behavior of exploring, exploiting, and conquering nature by human beings. The optimization methods are proposed to solve them. But unfortunately, because of the no free lunch rule [1], it is always hard to find a universal efficient way for almost all problems. Therefore, scientists and engineers around the world are still under ways to find more optimization algorithms and more suitable methods.

Traditionally, the optimization algorithms are divided into two parts: the deterministic algorithms and the stochastic algorithms [2]; the deterministic algorithms are proved to be easily trapped in local optima, while the stochastic algorithms are found to be capable of avoiding local solutions with randomness. Thus, more attention is paid to the stochastic algorithms, and more and more algorithms are proposed. Among the research on the stochastic algorithms, presentations, improvements, and applications of the nature-inspired computing (NIC) algorithms come into being a hot spot.

The NIC algorithms are proposed with inspiration of the nature, and they have been proved to be efficient to solve the problems human meet [3, 4]. One of the most important parts of NIC algorithms are the bionic algorithms, and most of the bionic algorithms are metaheuristic [5–7]. They can solve problems with parallel computing and global searching. The metaheuristic algorithms divide the swarms in global and local searching with some methods. They cannot guarantee the global optimal solutions; thus, most of the metaheuristic algorithms introduce randomness to avoid local optima. The individuals in swarms are controlled to separate, align, and cohere [8] with randomness; their current velocities are composed of the former velocities, random multipliers of the frequency [9], or Euclidean distances of specific individuals' positions [10–14]. Some improvements are made with inertia weights modification [15–17], hybridization with invasive weed optimization [18], chaos [19], and binary [20] vectors et al. Most of these improvements result in a little better performance of the specific algorithms, but the overall structures remain unchanged.

Almost all of the metaheuristic algorithms and their improvements so far are inspired directly from the behaviors of the organisms such as searching, hunting [11, 21], pollinating [13], and flashing [14]. In the old metaheuristic algorithms, such as the genetic algorithm (GA) [22], simulated annealing (SA) [23], and the ant colony optimization (ACO) algorithm [24], the individuals are treated in the same way, and the final results are the best fitness values. Metaheuristic algorithms perform their behavior under the same governing equations. To achieve a better performance and decrease the possibility of being trapped in local optima, random walks or levy flights are introduced to the individuals when specific conditions are might [25, 26]. These mostly mean that the swarms would perform their behavior in more uncontrolling ways. Furthermore, as organisms living in swarms in nature, most of them have social hierarchies as long as they are slightly intelligent. For example, in an ant colony, the queen is the commander despite its reproduction role; the dinergates are soldiers to garden the colony, while the ergates are careered with building, gathering, and breeding. It can be concluded that the hierarchy of the ant colony is queen → dinergates → ergates if they are classified with jobs. The ergates' behavior could be directed by their elder's experience and their queen or the dinergates. If the ergates are commanded by the queen, some dinergates, or elders, and such operations are mathematically described and introduced to the ant colony optimization (ACO) in some way, will the ACO algorithm perform better in solving the problems? In other words, how about the social hierarchy of the swarms considered in the metaheuristic algorithms? This work was done by Mirjalili et al., and a new optimization method called the grey wolf optimization (GWO) algorithm was proposed [27].

The GWO algorithm considers the searching, hunting behavior, and the social hierarchy of the grey wolves. Due to less randomness and varying numbers of individuals assigned in global and local searching procedures, the GWO algorithm is easier to use and converges more rapidly. It has been proved to be more efficient than the PSO [27] algorithm and other bionic algorithms [28–32]. More attention had been paid to its applications due to its better performance. Efforts have been done in feature and band selection [33, 34], automatic control [29, 35], power dispatching [32, 36], parameter estimation [31], shop scheduling [28], and multiobjective optimization [37, 38]. However, the standard GWO algorithm was formulated with equal importance of the grey wolves' positions, which is not consistent strictly with their social hierarchy. Recent developments of the GWO algorithms such as the binary GWO algorithm [34], multiobjective GWO algorithm [37], and mix with others [39], together with their applications [40–43] keep it remaining unchanged. If the searching and hunting positions of the grey wolves are also agreed to the social hierarchy, the GWO algorithm will be possibly improved. With a hypothesis that the social hierarchy of the grey wolves would be also functional in the grey wolves' searching procedure, we report an improvement of the original GWO algorithm in this paper. And considering the applications in engineering when a maximum admissible error (MAE) is usually restricted for given problems, a declined exponentially governing equation of the controlling parameter is introduced to avoid the unknown maximum iteration number. The rest of this paper is organized as follows:

Section 2 presents the inspiration of the improvement and the revision of the controlling equations to meet the needs of the latter experiments. Experiment setup is described in Section 3, and results are compared in Section 4. Finally, Section 5 concludes the work and further research suggestions are made.

## 2. Algorithms

According to Mirjalili et al. [27], the grey wolves live together and hunt in groups. The searching and hunting process can be described as follows: (1) if a prey is found, they first track and chase and approach it. (2) If the prey runs, then the grey wolves pursue, encircle, and harass the prey until it stops moving. (3) Finally, the attack begins.

### 2.1. Standard GWO Algorithm

Mirjalili designed the optimization algorithm imitating the searching and hunting process of grey wolves. In the mathematical model, the fittest solution is called the alpha (*α*), the second best is beta (*β*), and consequently, the third best is named the delta (*δ*). The rest of the candidate solutions are all assumed to be omegas (*ω*). All of the omegas would be guided by these three grey wolves during the searching (optimizing) and hunting.

When a prey is found, the iteration begins (*t* = 1). Thereafter, the alpha, beta, and the delta wolves would lead the omegas to pursue and eventually encircle the prey. Three coefficients $\vec{A}$, $\vec{C}$, and $\vec{D}$ are proposed to describe the encircling behavior:

(1) $$\begin{array}{c} \vec{D_{\alpha }}=\left|\vec{C_{1}}\cdot \vec{X_{a}}-\vec{X}\left(t\right)\right|,\\ \vec{D_{\beta }}=\left|\vec{C_{2}}\cdot \vec{X_{\beta }}-\vec{X}\left(t\right)\right|,\\ \vec{D_{\delta }}=\left|\vec{C_{3}}\cdot \vec{X_{\delta }}-\vec{X}\left(t\right)\right|, \end{array}$$

where *t* indicates the current iteration, $\vec{X}$ is the position vector of the grey wolf, and $\vec{X_{1}}$, $\vec{X_{2}}$, and $\vec{X_{3}}$ are the position vectors of the alpha, beta, and delta wolves. $\vec{X}$ would be computed as follows:

(2) $$\begin{array}{c} \vec{X_{1}}=\vec{X_{a}}-\vec{A_{1}}\cdot \vec{D_{\alpha }}, \end{array}$$

(3) $$\begin{array}{c} \vec{X_{2}}=\vec{X_{\beta }}-\vec{A_{2}}\cdot \vec{D_{\beta }}, \end{array}$$

(4) $$\begin{array}{c} \vec{X_{3}}=\vec{X_{\delta }}-\vec{A_{3}}\cdot \vec{D_{\delta }}, \end{array}$$

(5) $$\begin{array}{c} \vec{X}\left(t\right)=\frac{\vec{X_{1}}+\vec{X_{2}}+\vec{X_{3}}}{3}. \end{array}$$

The parameters $\vec{A}$ and $\vec{C}$ are combinations of the controlling parameter *a* and the random numbers $\vec{r_{1}}$ and $\vec{r_{2}}$ [27]:

(6) $$\begin{array}{c} \vec{A}=2\alpha \vec{r_{1}}-\alpha ,\\ \vec{C}=2\vec{r_{2}}. \end{array}$$

The controlling parameter *a* changes $\vec{A}$ and finally causes the omega wolves to approach or run away from the dominant wolves such as the alpha, beta, and delta. Theoretically, if $\left|\vec{A}\right|>1$, the grey wolves run away from the dominants, and this means the omega wolves would run away from the prey and explore more space, which is called a global search in optimization. And if $\left|\vec{A}\right|<1$, they approach the dominants, which means the omega wolves would follow the dominants approaching the prey, and this is called a local search in optimization.

The controlling parameter *a* is defined to be declined linearly from a maximum value of 2 to zero while the iterations are being carried on:

(7) $$\begin{array}{c} \alpha =2\left(1-\frac{\text{it}}{N}\right), \end{array}$$

where *N* is the maximum iteration number, and it is initialized at the beginning by users. It is defined as the cumulative iteration number. The application procedure can be divided in three parts. (1) The given problems are understood and mathematically described, and some elemental parameters are then known. (2) A pack of grey wolves are randomly initialized all through the space domain. (3) The alpha and other dominant grey wolves lead the pack to search, pursue, and encircle the prey. When the prey is encircled by the grey wolves and it stops moving, the search finishes and attacks begin. The pseudocode is listed in Table 1.

### 2.2. Proposed Variable Weights and Their Governing Equations

We can see from the governing equation (5) that the dominants play a same role in the searching process; every one of the grey wolves approaches or runs away from the dominants with an average weight of the alpha, beta, and delta. However, although the alpha is the nearest to the prey at the beginning of the search, it might be far away from the final result, let alone the beta and delta. Therefore, at the beginning of the searching procedure, only the position of the alpha should be considered in equation (5), or its weight should be much larger than those of other dominants. On the contrary, the averaging weight in equation (5) is also against the social hierarchy hypothesis of the grey wolves. If the social hierarchy is strictly followed in the pack, the alpha is the leader and he/she might be always the nearest one to the prey. The alpha wolf should be the most important, which means that the weight of alpha's position in equation (5) should be always no less than those of the beta and the delta. And consequently, the weight of the beta's position should be always no less than that of the delta. Based on these considerations, we further hypothesize the following:

1. The searching and hunting process are always governed by the alpha, the beta plays a less important role, and the delta plays a much less role. All of the other grey wolves transfer his/her position to the alpha if he/she gets the best.
2. It should be noted that, in real searching and hunting procedures, the best position is nearest to the prey, while in optimization for a global optimum of a given problem, the best position is the maximum or minimum of the fitness value under given restrictions.
3. During the searching process, a hypothesized prey is always surrounded by the dominants, while in hunting process, a real prey is encircled. The dominant grey wolves are at positions surrounding the prey in order of their social hierarchy. This means that the alpha is the nearest one among the grey wolves; the beta is the nearest one in the pack except for the alpha; and the delta ranks the third. The omega wolves are involved in the processes, and they transfer their better positions to the dominants.

With hypothesis mentioned hereinbefore, the update method of the positions should not be considered the same in equation (5).

When the search begins, the alpha is the nearest, and the rest are all not important. So, his/her position should be contributed to the new searching individuals, while all of the others could be ignored. This means that the weight of the alpha should be near to 1.0 at the beginning, while the weights of the beta and delta could be near zero at this time. At the final state, the alpha, beta, and the delta wolves should encircle the prey, which means they have an equal weight, as mentioned in equation (5). Along with the searching procedure from the beginning to the end, the beta comes up with the alpha as he/she always rank the second, and the delta comes up with the beta due to his/her third rank. This means that the weights of the beta and delta arise along with the cumulative iteration number. So, the weight of the alpha should be reduced, and the weights of the beta and delta arise.

The above ideas could be formulated in mathematics. First of all, all of the weights should be varied and limited to 1.0 when they are summed up. Equation (5) is then changed as follows:

(8) $$\begin{array}{c} \vec{X}\left(t+1\right)=w_{1}\vec{X_{1}}+w_{2}\vec{X_{2}}+w_{3}\vec{X_{3}},\\ w_{1}+w_{2}+w_{3}=1. \end{array}$$

Secondly, the weight of the alpha *w*_1_, that of the beta *w*_2_, and that of the delta *w*_3_, should always satisfy *w*_1_ ≥ *w*_2_ ≥ *w*_3_. Mathematically speaking, the weight of the alpha would be changed from 1.0 to 1/3 along with the searching procedure. And at the same time, the weights of the beta and delta would be increased from 0.0 to 1/3. Generally speaking, a cosine function could be introduced to describe w_1_ when we restrict an angle *θ* to vary in [0, arccos(1/3)].

Thirdly, the weights should be varied with the cumulative iteration number or “it”. And we know that *w*_2_ · *w*_3_⟶0 when it = 0 and *w*_1_, *w*_2_, *w*_3_⟶1/3 when it ⟶ ∞. So, we introduce an arc-tangent function about it which would be varying from 0.0 to *π*/2. And magically sin (*π*/4) = cos (*π*/4) = $\sqrt{2}/2$, so another angular parameter *φ* was introduced as follows:

(9) $$\begin{array}{c} \varphi =\frac{1}{2}\arctan\left(\text{it}\right). \end{array}$$

Considering *w*_2_ would be increased from 0.0 to 1/3 along with *it*, we hypothesize that it contains sin *θ* and cos *φ* and *θ* ⟶ arccos(1/3) when it ⟶ ∞; therefore,

(10) $$\begin{array}{c} \theta =\frac{2}{\pi }\arccos\frac{1}{3}\cdot \arctan\left(\text{it}\right), \end{array}$$

when it ⟶ ∞, *θ* ⟶ arccos (1/3), *w*_2_ = 1/3, we can then formulate *w*_2_ in details. Based on these considerations, a new update method of the positions with variable weights is proposed as follows:

(11) $$\begin{array}{c} w_{1}=\cos\theta ,\\ w_{2}=\frac{1}{2}\sin\theta \;\cdot \;\cos\varphi ,\\ w_{3}=1-w_{1}-w_{2}. \end{array}$$

**Figure 1:**
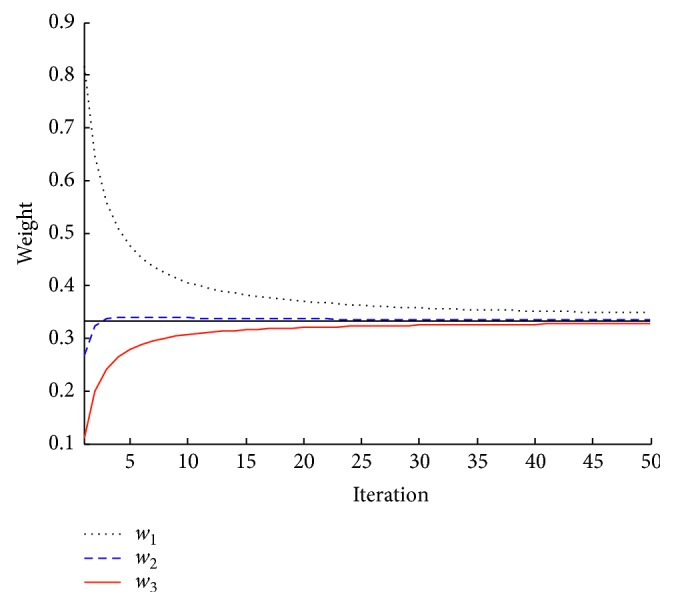
The variable weights vs. iterations.

The curve of the variable weights is drawn in Figure 1. We can then find that the variable weights satisfy the hypothesis, the social hierarchy of the grey wolves' functions in their behavior of searching.

### 2.3. Proposed Declined Exponentially Governing Equation of the Controlling Parameter

In equation (7), the controlling parameter is declined linearly from two to zero when the iterations are carrying on from zero to the maximum *N*. However, an optimization is usually ended with a maximum admissible error (MAE) which is requested in engineering. This also means that the maximum iteration number *N* is unknown.

Furthermore, the controlling parameter is a restriction parameter for A, who is responsible for the grey wolf to approach or run away from the dominants. In other words, the controlling parameter governs the grey wolves to search globally or locally in the optimizing process. The global search probability is expected to be larger when the search begins; and consequently, the local search probability is expected to be larger when the algorithm is approaching the optimum. Therefore, to obtain a better performance of the GWO algorithm, the controlling parameter is expected to be decreased quickly when the optimization starts and converge to the optimum very fast. On the contrary, some grey wolves are expected to remain global searching to avoid being trapped in local optima. Considering these reasons, a controlling parameter declined exponentially [44] is introduced as described below:

(12) $$\begin{array}{c} \alpha =\alpha_{m}e^{-\text{it}/M}, \end{array}$$

where *a*_*m*_ is the maximum value and *M* is an admissible maximum iteration number. The parameter *M* restricts the algorithm to avoid long time running and nonconvergence. It is expected to be larger than 10^4^ or 10^5^ based on nowadays computing hardware used in most laboratories.

## 3. Empirical Studies and the Experiments Prerequisite

The goal of experiments is to verify the advantages of the improved GWO algorithm with variable weights (VW-GWO) with comparisons to the standard GWO algorithm and other metaheuristic algorithms in this paper. Classically, optimization algorithms are applied to optimize benchmark functions which were used to describe the real problems human meet.

### 3.1. Empirical Study of the GWO Algorithm

**Figure 2:**
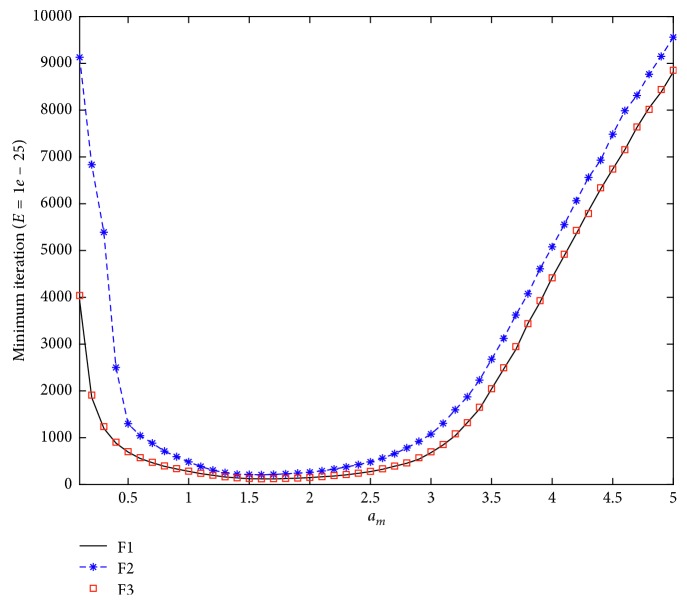
Relationship between the MLIT and maximum value *a*_*m*_.

Although there are less numbers of parameters in the GWO algorithm than that in other algorithms such as the ALO, PSO, and bat algorithm (BA) [45], the suitable values of the parameters remain important for the algorithm to be efficient and economic. Empirical study has been carried out, and results show that the population size is expected to be 20∼50 balancing the computing complexity and the convergent rate. In an empirical study on the parameters of the maximum value *a*_*m*_, the sphere function (F1) and Schwefel's problems 2.22 (F2) and 1.2 (F3) are optimized to find the relationship between *a*_*m*_ and the mean least iteration times with a given error tolerance of 10^−25^, as shown in Figure 2.

We can know from Figure 2 the following: (1) the maximum value *a*_*m*_ of the controlling parameter *a* influences the MLIT under a given MAE; when *a*_*m*_ is smaller than 1.0, the smaller the *a*_*m*_ is, the more the MLIT would be needed. On the contrary, if the *a*_*m*_ is larger than 2.5, the larger the *a*_*m*_ is, the more the MLIT would be needed. (2) *a*_*m*_ should be varied in [1.0, 2.5], and *a*_*m*_ is found to be the best when it is 1.6 or 1.7.

### 3.2. Benchmark Functions

Benchmark functions are standard functions which are derived from the research on nature. They are usually diverse and unbiased, difficult to be solved with analytical expressions. The benchmark functions have been an essential way to test the reliability, efficiency, and validation of optimization algorithms. They varied from the number of ambiguous peaks in the function landscape, the shape of the basins or valleys, reparability to the dimensional. Mathematically speaking, the benchmark functions can be classified with the following five attributes [46].

- Continuous or uncontinuous: most of the functions are continuous, but some of them are not.
- Differentiable or nondifferentiable: some of the functions can be differenced, but some of them not.
- Separable or nonseparable: some of the functions can be separated, but some of them are not.
- Scalable or nonscalable: some of the functions can be expanded to any dimensional, but some of them are fixed to two or three dimensionalities.
- Unimodal or multimodal: some of the functions have only one peak in their landscape, but some of them have many peaks. The former attribute is called unimodal, and the latter is multimodal.

There are 175 benchmark functions, being summarized in literature [46]. In this paper, we choose 11 benchmark functions from simplicity to complexity including all of the above five characteristics. They would be fitted to test the capability of the involved algorithms, as listed in Table 2, and they are all scalable.

The functions are all *n*-dimensional, and their input vectors *x* = (*x*_1_, *x*_2_,…, *x*_*n*_) are limited by the domain. Values in the domain are maximum to be *ub* and minimum to be *lb*. The single result values are all zeros theoretically for simplicity.

## 4. Results and Discussion

There are 11 benchmark functions being involved in this study. Comparisons are made with the standard grey wolf optimization algorithm (std. GWO) and three other bionic methods such as the ant lion optimization algorithm (ALO), the PSO algorithm, and BA.

### 4.1. General Reviews of the Algorithms

The randomness is all involved in the algorithms studied in this paper, for example, the random positions, random velocities, and random controlling parameters. The randomness causes the fitness values obtained during the optimization procedure to fluctuate. So, when an individual of the swarm is initialized or it randomly jumps to a position quite near the optimum, the best fitness value would be met.  Table 3 lists the best and worst fitness results of some chosen benchmark functions and their corresponding algorithms. During this experiment, 100 Monte Carlo (MC) simulations are carried out for every benchmark function. The results show that the randomness indeed leads to some random work, but at most of the time, the final results would be more dependent on the algorithms.

**Figure 3:**
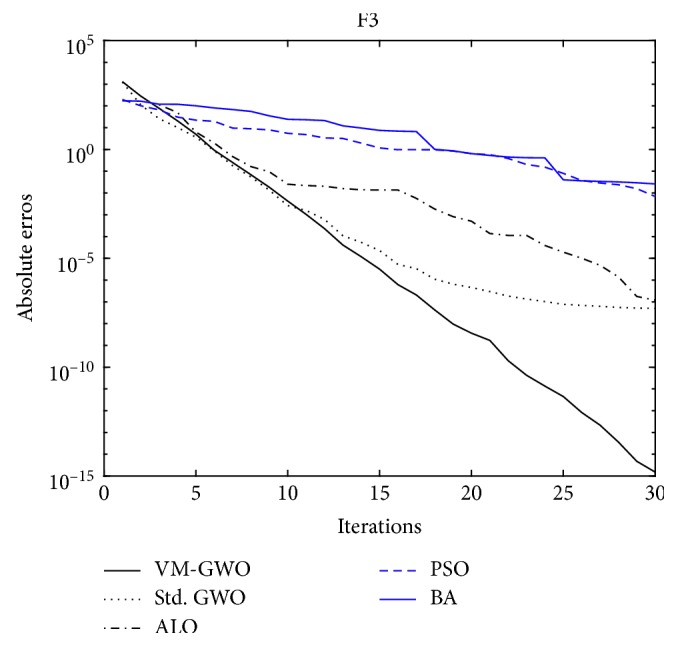
F3: convergence vs. iterations (dim = 2).

The GWO algorithms always work the best at first glance of Table 3, either the VM-GWO or the std. GWO algorithm could optimize the benchmark functions best to its optima with little absolute errors, while the proposed VM-GWO algorithm is almost always the best one. Other compared algorithms such as the PSO, ALO algorithms, and the BA would lead to the worst results at most time. These mean that the GWO algorithms are more capable, and the proposed VM-GWO algorithm is indeed improving the capability of the std. GWO algorithm. A figure about the absolute errors averaged over MC = 100 versus iterations could also lead to this conclusion, as shown in Figure 3.

The convergence rate curve during the iterations of F3 benchmark function is demonstrated in Figure 3. It shows that the proposed VM-GWO algorithm would result in faster converging, low residual errors, and stable convergence.

### 4.2. Comparison, Statistical Analysis, and Test

General acquaintances of the metaheuristic algorithms might be got from Table 3 and Figure 3. However, the optimization problems often demand the statistical analysis and test. To do this, 100 MC simulations are carried out on the benchmark functions. The benchmark functions are all two dimensional, and they are optimized by the new proposed VM-GWO and other four algorithms over 100 times. Causing the benchmark functions are all concentrated to zeros, and the simulated fitness results are also their absolute errors. The mean values of the absolute errors and the standard deviations of the final results are listed in Table 4; some of the values are quoted from the published jobs, and references are listed correspondingly.

The proposed VM-GWO algorithm and its compared algorithms are almost all capable of searching the global optima of the benchmark functions. The detailed values in Table 4 show that the standard deviations of the 100 MC simulations are all small. We can further draw the following conclusions:

1. All of the algorithms involved in this study were able to find the optimum.
2. All of the benchmark functions tested in this experiment could be optimized, whether they are unimodal or multimodal, under the symmetric or unsymmetric domain.
3. Comparatively speaking, although the bat algorithm is composed of much more randomness, it did the worst job. The PSO and the ALO algorithm did a little better.
4. The GWO algorithms implement the optimization procedure much better. The proposed VM-GWO algorithm optimized most of the benchmark functions involved in this simulation at the best, and it did much better than the standard algorithm.

Therefore, the proposed VM-GWO algorithm is better performed in optimizing the benchmark functions than the std. GWO algorithm as well as the ALO, PSO algorithm, and the BA, which can be also obtained from the Wilcoxon rank sum test [47] results, as listed in Table 5.

In Table 5, the *p* values of the Wilcoxon rank sum test is reported and show that the proposed VM-GWO algorithm has superiority over most of the benchmark functions except F5: Rosenbrock function.

### 4.3. Mean Least Iteration Times (MLIT) Analysis over Multidimensions

Compared with other bionic algorithms, the GWO algorithm has fewer numbers of parameter. Compared with the std. GWO algorithm, the proposed VM-GWO algorithm does not generate additional uncontrolling parameters. It furthermore improves the feasibility of the std. GWO algorithm by introducing an admissible maximum iteration number. On the contrary, there are large numbers of randomness in the compared bionic algorithms such as the ALO, PSO algorithms, and the BA. Therefore, the proposed algorithm is expected to be fond by the engineers, who need the fastest convergence, the most precise results, and which are under most control. Thus, there is a need to verify the proposed algorithm to be fast convergent, not only a brief acquaintance from Figure 3.

Generally speaking, the optimization algorithms are usually used to find the optima under constrained conditions. The optimization procedure must be ended in reality, and it is expected to be as faster as capable. The admissible maximum iteration number *M* forbids the algorithm to be run endlessly, but the algorithm is expected to be ended quickly at the current conditions. This experiment will calculate the mean least iteration times (MLIT) under a maximum admissible error. The absolute values of MAE are constrained to be less than 1.0 × 10^−3^ and *M* = 1.0 × 10^5^. In this experiment, 100 MC simulations are carried out, and for simplicity, not all classical benchmark functions are involved in this experiment. The final statistical results are listed in Tables 6 [Table tab7]–8. Note that the complexity of the ALO algorithm is very large, and it is time exhausted based on the current simulation hardware described in Appendix. So, it is not included in this experiment.

Table 8 lists the MLIT data when VW-GWO, std. GWO, PSO algorithm, and BA are applied to the unimodal benchmark function F1. The best, worst, and the standard deviation MLIT values are listed. The mean values are also calculated, and *t*-tested are carried out with *α* = 0.05. The last column lists the remaining MC simulation numbers discarding all of the data when the searching processes reach the admissible maximum iteration number *M*. The final results demonstrate the best performance of the proposed VM-GWO algorithm on unimodal benchmark functions compared to other four algorithms involved. The data in Tables 6 [Table tab7]–8 are under the same conditions, and only difference is that Table 6 lists the data obtained when the algorithms are applied to a multimodal benchmark function with the symmetrical domain. However, Table 8 lists the data obtained when the algorithms applied to a multimodal benchmark function with the unsymmetrical domain. A same conclusion could be drawn.

Note that, in this experiment, the dimensions of the benchmark functions are varied from 2 to 10 and 30. The final results also show that if the dimensions of the benchmark functions are raised, the MLIT values would be increased dramatically. This phenomenon would lead to the doubt whether it also performs the best and is capable to solve high-dimensional problems.

### 4.4. High-Dimensional Availability Test

Tables 6 [Table tab7]–8 show that the larger the dimensions are, the more the MLIT values would be needed to meet to experiment constraints. However, as described in the first part, the optimization algorithms are mostly developed to solve the problems with huge number of variables, massive complexity, or having no analytical solutions. Thus, the high-dimensional availability is quite interested. As described in the standard GWO algorithm, the proposed VM-GWO algorithm should also have the merits to solve the large-scale problems. An experiment with dim = 200 is carried out to find the capability of the algorithms solving the high-dimensional problems. For simplicity, three classical benchmark functions, such as F4: Schwefel's problem 2.21 function, F8: exponential function, and F11: Zakharov function, are used to demonstrate the results, as listed in Table 9. The final results of 100 MC experiments will be evaluated and counted, and each time the search procedure will be also iterated for a hundred times.

The data listed in Table 9 show that the GWO algorithms would be quickly convergent, and the proposed algorithm is the best to solve the large-scale problems.

**Figure 4:**
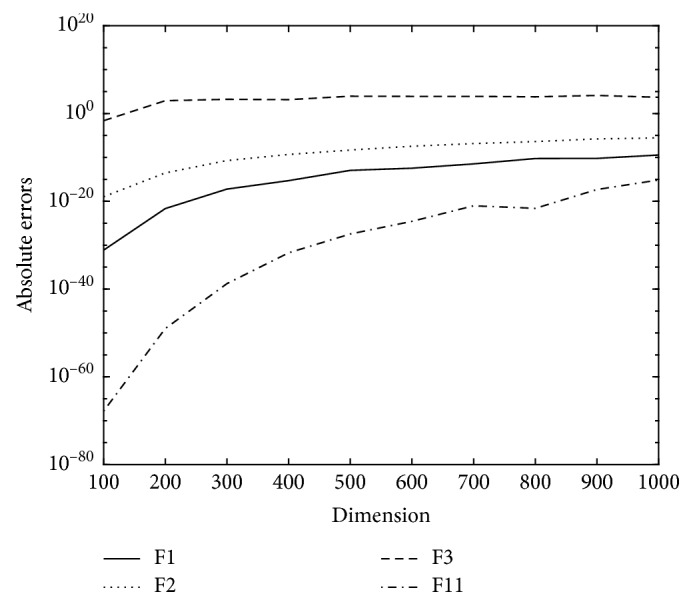
Absolute errors vs. dimensions based on VM-GWO.

To test its capability even further, we also carry out an experiment to verify the capability solving some benchmark function in high dimensions with restrictions MC = 100 and MLIT = 500. In this experiment, we change the dimensions from 100 to 1000, and the final results which are also the absolute errors averaged over MC times, being shown in Figure 4.

We can see from Figure 4 that the VM-GWO is capable to solve high-dimensional problems.

## 5. Conclusions

In this paper, an improved grey wolf optimization (GWO) algorithm with variable weights (VW-GWO algorithm) is proposed. A hypothesize is made that the social hierarchy of the packs would also be functional in their searching positions. And variable weights are then introduced to their searching process. To reduce the probability of being trapped in local optima, a governing equation of the controlling parameter is introduced, and thus, it is declined exponentially from the maximum. Finally, three types of experiments are carried out to verify the merits of the proposed VW-GWO algorithm. Comparisons are made to the original GWO and the ALO, PSO algorithm, and BA.

All the selected experiment results show that the proposed VW-GWO algorithm works better under different conditions than the others. The variance of dimensions cannot change its first position among them, and the proposed VW-GWO algorithm is expected to be a good choice to solve the large-scale problems.

However, the proposed improvements are mainly focusing on the ability to converge. It leads to faster convergence and wide applications. But it is not found to be capable for all the benchmark functions. Further work would be needed to tell the reasons mathematically. Other initializing algorithms might be needed to let the initial swarm individuals spread all through the domain, and new searching rules when the individuals are at the basins would be another hot spot of future work.

## Figures and Tables

**Table 1:** Pseudocode of the GWO algorithm.

Description	Pseudocode
Set up optimization	Dimension of the given problems
	Limitations of the given problems
	Population size
	Controlling parameter
	Stop criterion (maximum iteration times or admissible errors)

Initialization	Positions of all of the grey wolves including *α*, *β*, and *δ* wolves

Searching	While not the stop criterion, calculate the new fitness function
	Update the positions
	Limit the scope of positions
	Refresh *α*, *β*, and *δ*
	Update the stop criterion
	End

**Table 2:** Benchmark functions to be fitted.

Label	Function name	Expressions	Domain [*lb*, *ub*]
F1	De Jong's sphere	*y*=∑_*i*=1_^*n*^ *x*_*i*_^2^	[−100, 100]
F2	Schwefel's problems 2.22	*y*=∑_*i*=1_^*n*^ *⌊x*_*i*_ *⌋*+∏_*i*=1_^*n*^ *⌊x*_*i*_ *⌋*	[−100, 100]
F3	Schwefel's problem 1.2	*y*=∑_*i*=1_^*n*^∑_*j*=1_^*i*^ *x*_*j*_^2^	[−100, 100]
F4	Schwefel's problem 2.21	$y=\underset{1\leq i\leq n}{\max}\lfloor x_{i}\rfloor$	[−100, 100]
F5	Chung Reynolds function	*y*=(∑_*i*=1_^*n*^ *x*_*i*_^2^)^2^	[−100, 100]
F6	Schwefel's problem 2.20	*y*=∑_*i*=1_^*n*^ *⌊x*_*i*_ *⌋*	[−100, 100]
F7	Csendes function	*y*=∑_*i*=1_^*n*^ *x*_*i*_^2^(2+sin(1/*x*_*i*_))	[−1, 1]
F8	Exponential function	*y*=−*e* ^−0.5∑_*i*=1_^*n*^*x*_*i*_^2^^	[−1, 1]
F9	Griewank's function	$y=\sum_{i=1}^{n}\left(x_{i}^{2}/4000\right)-\prod_{i=1}^{n}\cos\left(x_{i}/\sqrt{i}\right)+1$	[−100, 100]
F10	Salomon function	$y=1-\cos\left(2\pi \sqrt{\sum_{i=1}^{n}x_{i}^{2}}\right)+0.1\sqrt{\sum_{i=1}^{n}x_{i}^{2}}$	[−100, 100]
F11	Zakharov function	*y*=∑_*i*=1_^*n*^ *x*_*i*_^2^+(∑_*i*=1_^*n*^0.5*ix*_*i*_)^2^+(∑_*i*=1_^*n*^0.5*ix*_*i*_)^4^	[−5, 10]

**Table 3:** The best and worst simulation results and their corresponding algorithms (dim = 2).

Functions	Value	Corresponding algorithm
*Best fitness*		
F1	1.4238*e* − 70	VM-GWO
F2	3.2617*e* − 36	VM-GWO
F3	3.6792*e* − 68	VM-GWO
F4	3.3655*e* − 66	Std. GWO
F7	7.8721*e* − 222	VM-GWO
F8	0	VM-GWO, Std. GWO, PSO, BA
F9	0	VM-GWO, Std. GWO
F11	2.6230*e* − 69	VM-GWO

*Worst fitness*		
F1	1.0213*e* − 07	BA
F2	4.1489*e* − 04	BA
F3	5.9510*e* − 08	BA
F4	2.4192*e* − 06	PSO
F7	1.0627*e* − 24	BA
F8	5.7010*e* − 13	BA
F9	1.0850*e* − 01	ALO
F11	9.9157*e* − 09	BA

**Table 4:** Statistical analysis on the absolute errors of the selected functions (dim = 2).

Functions	VM-GWO		Std. GWO		ALO		PSO		BA	
	Mean	Std. deviation	Mean	Std. deviation	Mean	Std. deviation	Mean	Std. deviation	Mean	Std. deviation
F1	7.2039*e* − 66	3.5263*e* − 65	6.59*E* − 28	6.34*E* − 5 [27]	2.59*E* − 10	1.65*E* − 10 [2]	1.36*E* − 4	2.02*E* − 4 [27]	0.773622	0.528134 [2]
F2	1.3252*e* − 34	3.5002*e* − 34	7.18*E* − 17	0.02901 [27]	1.84241*E* − 6	6.58*E* − 7 [2]	0.042144	0.04542 [27]	0.334583	3.186022 [2]
F3	3.7918*e* − 60	1.1757*e* − 59	3.29*E* − 6	79.1496 [27]	6.0685*E* − 10	6.34*E* − 10 [2]	70.12562	22.1192 [27]	0.115303	0.766036 [2]
F4	2.2262*e* − 46	2.8758*e* − 46	5.61*E* − 7	1.31509 [27]	1.36061*E* − 8	1.81*E* − 9 [2]	0.31704	7.3549 [27]	0.192185	0.890266 [2]
F5	3.6015*e* − 131	9.0004*e* − 131	7.8319*e* − 97	2.4767*e* − 96	2.1459*e* − 20	2.8034*e* − 20	8.4327*e* − 20	1.7396*e* − 19	1.7314*e* − 17	4.9414*e* − 17
F9	0.0047	0.0040	0.00449	0.00666 [27]	0.0301	0.0329	0.00922	0.00772 [27]	0.0436	0.0294
F10	0.0200	0.0421	0.0499	0.0526	0.01860449	0.009545 [2]	0.273674	0.204348 [2]	1.451575	0.570309 [2]
F11	1.2999*e* − 60	4.1057*e* − 60	6.8181*e* − 35	1.5724*e* − 34	1.1562*e* − 13	1.2486*e* − 13	2.3956*e* − 12	3.6568*e* − 12	5.0662*e* − 09	4.9926*e* − 09

**Table 5:** *p* values of the Wilcoxon rank sum test for VM-GWO over benchmark functions (dim = 2).

	F1	F2	F3	F4	F5	F6	F7	F8	F9	F10	F11
Std. GWO	0.000246	0.00033	0.000183	0.00044	0.000183	0	0.000183	—	0.466753	0.161972	0.000183
PSO	0.000183	0.000183	0.000183	0.000183	0.472676	0	0.000183	0.167489	0.004435	0.025748	0.000183
ALO	0.000183	0.000183	0.000183	0.000183	0.472676	0	0.000183	0.36812	0.790566	0.025748	0.000183
BA	0.000183	0.000183	0.000183	0.000183	0.000183	0	0.000183	0.000747	0.004435	0.01133	0.000183

**Table 6:** MLITs and statistical results for F1.

dim	Algorithm	Best	Worst	Mean	*t*-test (*α* = 0.05)	Std. deviation	Number
2	VW-GWO	6	12	9.90	1.7180*e* − 193	1.0493	100
	Std. GWO	7	13	10.38	1.8380*e* − 22	1.2291	100
	PSO	48	1093	357.97	3.2203*e* − 22	205.3043	100
	BA	29	59	41.00	1.3405*e* − 101	5.8517	100

10	VW-GWO	53	66	59.97	4.1940*e* − 177	2.7614	100
	Std. GWO	74	89	80.40	1.9792*e* − 80	2.7614	100
	PSO	5713	11510	9279.22	2.9716*e* − 76	1300.8485	88
	BA	6919	97794	44999.04	7.5232*e* − 26	25133.3096	78

30	VW-GWO	55	67	59.85	1.2568*e* − 122	2.4345	100
	Std. GWO	71	86	80.07	2.6197*e* − 79	3.3492	100
	PSO	5549	12262	9314.78	9.6390*e* − 83	1316.3384	96
	BA	7238	92997	44189.16	5.2685*e* − 26	24831.7443	79

**Table 7 tab7:** MLITs and statistical results for F7.

dim	Algorithm	Best	Worst	Mean	*t*-test (*α* = 0.05)	Std. deviation	Number
2	VW-GWO	1	3	1.46	6.3755*e* − 226	0.5397	100
	Std. GWO	1	2	1.41	1.0070*e* − 229	0.4943	100
	PSO	2	2	2.00	0	0	100
	BA	1	3	1.02	8.5046*e* − 269	0.200	100

10	VW-GWO	5	9	7.65	5.7134*e* − 199	0.9468	100
	Std. GWO	5	11	7.48	5.1288*e* − 191	1.1413	100
	PSO	4	65	24.23	1.6196*e* − 85	10.9829	100
	BA	13	49	25.29	5.9676*e* − 109	6.2366	100

30	VW-GWO	13	22	17.14	9.6509*e* − 167	1.7980	100
	Std. GWO	15	30	20.80	1.3043*e* − 148	2.6208	100
	PSO	54	255	133.32	5.7600*e* − 12	42.5972	100
	BA	40	101	62.68	1.8501*e* − 53	11.8286	100

**Table 8:** MLITs and statistical results for F11.

dim	Algorithm	Best	Worst	Mean	*t*-test (*α* = 0.05)	Std. deviation	Number
2	VW-GWO	3	9	6.63	5.6526*e* − 188	1.2363	100
	Std. GWO	4	10	6.66	3.5865*e* − 186	1.2888	100
	PSO	6	125	46.35	1.6006*e* − 37	26.0835	100
	BA	5	62	27.58	1.6166*e* − 83	11.0080	100

10	VW-GWO	10	200	65.57	2.8562*e* − 12	43.2281	100
	Std. GWO	14	246	68.68	2.6622*e* − 11	41.7104	100
	PSO	15	1356	231.74	1.2116*e* − 6	257.1490	94
	BA	15	214	113.19	5.1511*e* − 2	66.9189	100

30	VW-GWO	49	1179	312.24	1.2262*e* − 18	194.7643	100
	Std. GWO	65	945	294.45	3.1486*e* − 21	160.7119	100
	PSO	32	5005	1086.11	6.0513*e* − 13	980.3386	72
	BA	66	403	221.60	1.9072*e* − 51	40.5854	100

**Table 9:** Statistical analysis on the absolute errors of the selected functions (dim = 200).

Functions	VM-GWO		Std. GWO		ALO		PSO		BA	
	Mean	Std. deviation	Mean	Std. deviation	Mean	Std. deviation	Mean	Std. deviation	Mean	Std. deviation
F4	3.3556*e* − 53	8.7424*e* − 53	1.6051*e* − 46	2.2035*e* − 46	4.2333*e* − 07	2.9234*e* − 07	3.0178*e* − 07	6.5449*e* − 07	1.6401*e* − 07	2.1450*e* − 07
F8	0	0	0	0	3.3307*e* − 17	7.4934*e* − 17	1.1102*e* − 17	3.5108*e* − 17	1.4466*e* − 14	1.9684*e* − 14
F11	0.0115	0.0193	0.0364	0.0640	8.3831	10.3213	12.6649	13.0098	4.7528*e* + 16	2.8097*e* + 16

## Data Availability

The associate software of this paper could be downloaded from http://ddl.escience.cn/f/Erl2 with the access code: kassof.

## Appendix

The simulation platform, as described in Section 3.3, is run on an assembled desktop computer being configured as follows: CPU: Xeon E3-1231 v3; GPU: NVidia GeForce GTX 750 Ti; memory: DDR3 1866 MHz; motherboard: Asus B85-Plus R2.0; hard disk: Kingston SSD.
